# A Simple Method for Establishing Adherent *Ex Vivo* Explant Cultures from Human Eye Pathologies for Use in Subsequent Calcium Imaging and Inflammatory Studies

**DOI:** 10.1155/2014/232659

**Published:** 2014-09-04

**Authors:** Sofija Andjelic, Xhevat Lumi, Zoltán Veréb, Natasha Josifovska, Andrea Facskó, Marko Hawlina, Goran Petrovski

**Affiliations:** ^1^Eye Hospital, University Medical Centre, 1000 Ljubljana, Slovenia; ^2^Department of Ophthalmology, Faculty of Medicine, University of Szeged, Korányi fasor 10-11, Szeged 6720, Hungary; ^3^Stem Cells and Eye Research Laboratory, Department of Biochemistry and Molecular Biology, Apoptosis and Genomics Research Group of the Hungarian Academy of Sciences, University of Debrecen, Szeged 6720, Hungary

## Abstract

A novel, simple, and reproducible method for cultivating pathological tissues obtained from human eyes during surgery was developed using viscoelastic material as a tissue adherent to facilitate cell attachment and expansion and calcium imaging of cultured cells challenged by mechanical and acetylcholine (ACh) stimulation as well as inflammatory studies. Anterior lens capsule-lens epithelial cells (aLC-LECs) from cataract surgery and proliferative diabetic retinopathy (PDR) fibrovascular epiretinal membranes (fvERMs) from human eyes were used in the study. We hereby show calcium signaling in aLC-LECs by mechanical and acetylcholine (ACh) stimulation and indicate presence of ACh receptors in these cells. Furthermore, an *ex vivo* study model was established for measuring the inflammatory response in fvERMs and aLC-LECs upon TNFα treatment.

## 1. Introduction

Human eye disease modeling requires well established* ex vivo* cell cultures. Such cultures allow studying diseases of interest at a cellular level using multiple techniques. In addition, they provide possibility to grow primary human eye cells with the purpose of repairing a defect and eventually transplanting them back to the patient in an autologous or heterologous manner.

An important condition for growing* ex vivo* eye explant cultures is to have an adherent environment. We developed a simple method for attaching eye tissue explants to the surface of a Petri dish by using surgical grade viscoelastic material, otherwise routinely used in ophthalmic surgery [1].

Human anterior lens capsule-lens epithelial cells (aLC-LECs) from cataract surgery and fibrovascular epiretinal membranes (fvERM) from proliferative diabetic retinopathy (PDR) were cultured adherently under viscoelastic material. The single-layered LECs underlying the aLC are metabolically the most active part of the lens and are responsible for sustaining physiological health of the tissue. ERMs are a collection of cells and extracellular matrix that occur in the inner, vitreal surface of the central retina. They have contractile properties and can lead to visual disturbance and metamorphopsia (distorted vision) due to their effect on the underlying retina. FvERMs represent the final and devastating stage of PDR and form, due to heavy hypoxia, retinal ischemia and unbalanced glucose metabolism, the result of which is a state of chronic inflammation [2, 3].

Cells growing out of cultured aLCs and fvERM explants were studied functionally by examining intracellular calcium [Ca^2+^]_i_ signaling under adherent culture conditions. Calcium signaling plays an important role in the regulation of cell function, affecting every aspect of the cells' life and death [4]. We hereby show free [Ca^2+^]_i_ changes upon mechanical and acetylcholine (ACh) stimulation in cultured cells obtained from human aLCs under adherent conditions and indicate presence of ACh receptors in these cells. In addition, the inflammatory nature of fvERMs and aLC-LECs as well as their relation to tumor necrosis factor alpha (TNFα) and angiogenesis is addressed here.

## 2. Methods

### 2.1. Tissue Collection and Processing

All tissue collection complied with the guidelines of the Helsinki Declaration and was approved by the National Medical Ethics Committee of Slovenia; all patients signed an informed consent form before surgery which was performed at the Eye Hospital, University Medical Centre (UMC), Ljubljana, Slovenia. Altogether 11 patients were included in this study—6 cultures were analyzed for mechanical stimulation and 5 cultures for ACh stimulation, with the patients' age ranging from 70 to 92 years.

The aLC explants consisted of a monolayer of LECs attached to the basal lamina and were obtained from uneventful cataract surgeries due to progredient cataract. Lenses were dissected so that the aLCs (i.e., basal lamina and associated LECs) were isolated from the fiber cells that form the bulk of the lens. FvERMs were obtained from patients undergoing vitrectomy due to intravitreal hemorrhage in PDR.

All explants were obtained from single patients and were usually placed in a single dish accordingly. Immediately after isolation, the excised human eye explants were placed in sterile tubes filled with DMEM:F12 (D8437, Sigma-Aldrich, Ayrshire, UK), supplemented with 10% fetal calf serum (FCS) (PAA Laboratories GmbH, Pasching, Austria), and transported from the operating room to the research department in the same building. The explants were then transferred to empty cell culture glass bottom Petri dishes (Mattek Corp., Ashland, MA, USA; 3.5 cm in diameter) or tissue culture 12-well plates (TPP, Sigma, Germany) by using microdissecting tweezers (WPI by Dumont, Med.Biologie, Germany). The aLC explants were placed into the culture dish so that the concave side with the LECs was on the top and oriented upwards. The time of culturing ranged from 6 to 48 days.

### 2.2. Tissue Fixation/Adherence by Viscoelastic Material

**Figure 1:**
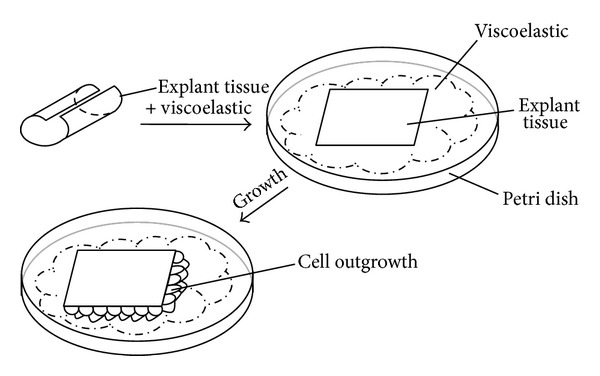
Method for adherent* ex vivo* cultivation of human eye tissue explants in a cell culture Petri dish.

For obtaining adherent conditions, careful removal of the remaining medium from the tissue cultures was performed by a micropipette, and then viscoelastic (HEALON OVD, Abbott Medical Optics, USA) was added on top of the explant to allow for flattening or “ironing” of the tissue onto the surface of the Petri dish (Figure 1).

For* ex vivo* cultivation under adherent conditions, DMEM:F12 supplemented with 10% FCS was then added slowly with the micropipette not to disturb or remove the viscoelastic cover on top of the explants. The micropipette tip was positioned close to the culture dish surface but far away from the explant, so that the medium arrived softly in contact with the viscoelastic and did not move the explant from its location. The culture dishes were then kept in a CO_2_ incubator (Innova CO-48; New Brunswick Scientific, Edison, NJ, USA) at 37°C and 5% CO_2_.

The culture dish was kept in the incubator without moving for 2-3 days in order to allow the cells to attach and start proliferating out of the explant. During medium change, the medium was removed gently and a fresh one was added subsequently by a micropipette from the opposite side of the explant in the dish, the pipette tip being close to the surface of the dish all the time. The viscoelastic dissolved over time and got replaced by new medium—time by which the explant was fully attached to the surface of the culture dish.

### 2.3. Light Microscopy and Calcium Imaging

The proliferation and migration of the cells were recorded throughout their continued growth using inverted light microscope (Axiovert S100, Carl Zeiss, AG, Oberkochen, Germany). The same microscope was used for [Ca^2+^]_i_ measurements. Image acquisition was carried out by a 12-bit cooled CCD camera SensiCam (PCO Imaging AG, Kelheim, Germany). The software used for the acquisition was WinFluor (written by J. Dempster, University of Strathclyde, Glasgow, UK). Microscope objectives used were 4x/0.10 Achroplan, 10x/0.30 Plan-Neofluar, 40x/0.50 LD A-plan, and 63x/1.25 oil Plan-Neofluar (Zeiss).

The excitation filters used were mounted on a Lambda LS-10 filter wheel (Sutter Instruments Co., CA, USA) and had a wavelength of 360 and 380 nm (Chroma Technology Corp., Bellows Falls, VT, USA). Excitation with the 360 nm filter (close to the Fura-2 isosbestic point) allowed observation of the cells' morphology and of the changes in the concentration of the dye, irrespective of the changes in [Ca^2+^]_i_, while the 360/380 nm ratio allowed visualization of the [Ca^2+^]_i_ changes in the cytoplasm. Image acquisition, timing, and filter wheel operation were all controlled by WinFluor software via a PCI6229 interface card (National Instruments, Austin, TX, USA). The light source used was XBO-75W (Zeiss) Xe arc lamp. The light intensity was attenuated when necessary with grey filters with optical densities 0.5, 1, and 2 (Chroma Technology Corp., Bellows Falls, VT, USA). The criteria for selecting the region for imaging were the presence of adherent cells and good cell morphology both assessed by observation of transilluminated and 360 nm fluorescence images. Individual image frames were acquired every 500 ms resulting in frame cycles being 1 second long (two wavelengths).

For [Ca^2+^]_i_ monitoring, the cell cultures were loaded with the acetoxymethyl (AM) ester of Fura-2 (Fura-2 AM, Invitrogen-Molecular Probes, Carlsbad, CA, USA), intracellular calcium indicator. For loading, Fura-2 AM in dimethyl sulfoxide (DMSO) was suspended in 3 mL of medium (high glucose medium with FBS) or physiological saline with (in mM) NaCl (131.8), KCl (5), MgCl2 (2), NaH2PO4 (0.5), NaHCO3 (2), CaCl2 (1.8), HEPES (10), glucose (10), pH 7.24 to the final working concentration of 2 *μ*M (aLC). The loading was done in the incubator at 37°C for 30 min (aLC). After loading, the cell cultures were washed twice for 7 min with the medium or physiological saline. The final working concentration of Fura-2 and the time of incubation/washing were larger for larger eye explants (it depended on the explant size).

Fura-2 dye has two excitation (absorption) peaks (340 and 380 nm), an isosbestic point at 360 nm and one emission peak at 510 nm. Its absorption and fluorescent properties change in accordance with Ca^2+^ binding (low [Ca^2+^]_i_—high absorption at 380 nm, high [Ca^2+^]_i_—high absorption at 340 nm while the absorption is not Ca^2+^ dependent at the isosbestic point of 360 nm). The absorptive properties of Fura-2 allow the use of ratio imaging (360/380 ratio), which considerably reduces the effects of uneven dye loading, leakage of the dye, and photobleaching as well as problems associated with measuring [Ca^2+^]_i_ in cells of unequal thickness.

### 2.4. Mechanical and Acetylcholine (ACh) Stimulation

To test responses to mechanical stimuli, a tip of a glass micropipette mounted on a MP-285 micromanipulator (Sutter, Novato, CA, USA) was used. Prior to use, the tip of the pipette was heat-polished until it rounded up.

The agonist acetylcholine (ACh; Sigma, USA) was applied in 10 *μ*M concentration, which was enough to induce >90% maximal [Ca^2+^]_i_ response, according to the data by Collison et al. [12]. The agonist application as well as its washout from the bath was driven simply by the hydrostatic pressure of a 35 cm of water column and controlled manually by a luer-lock stopcock (WPI) and applied through a polyethylene plastic tubing (inner diameter 2 mm), attached to the coarse micromanipulator. The excess bathing solution was removed by a suction line.

### 2.5. Secretion of Inflammatory Cytokines by ELISA

The expanded fvERM cells were plated onto 6-well plates at a density of 2 × 10^5^ cells per well in triplicates. Similar plating was carried out in case of the aLC-LECs until proper cell number was achieved for cytokine measurements. After 24 hrs, the medium was changed, and the cells were treated with 100 ng/mL recombinant human TNF*α* (Preprotech, Rocky Hill, NJ, USA) for additional 24 hours. The secreted cytokines, IL-6, and IL-8 were analyzed by commercial ELISA kit (R&D, Germany) according to the manufacturer's protocol. Three independent experiments were performed on three different outgrowing cells from both fvERM and aLC.

## 3. Results

### 3.1. Human Eye Explant Tissues Adhere to the Cell Culture Dish under a Gravitational Force of Viscoelastic Material

Novel, simple, and reproducible method for* ex vivo* cultivation of human explant tissues (aLCs and fvERMs) was established using viscoelastic material (Figure 1).

**Figure 2:**
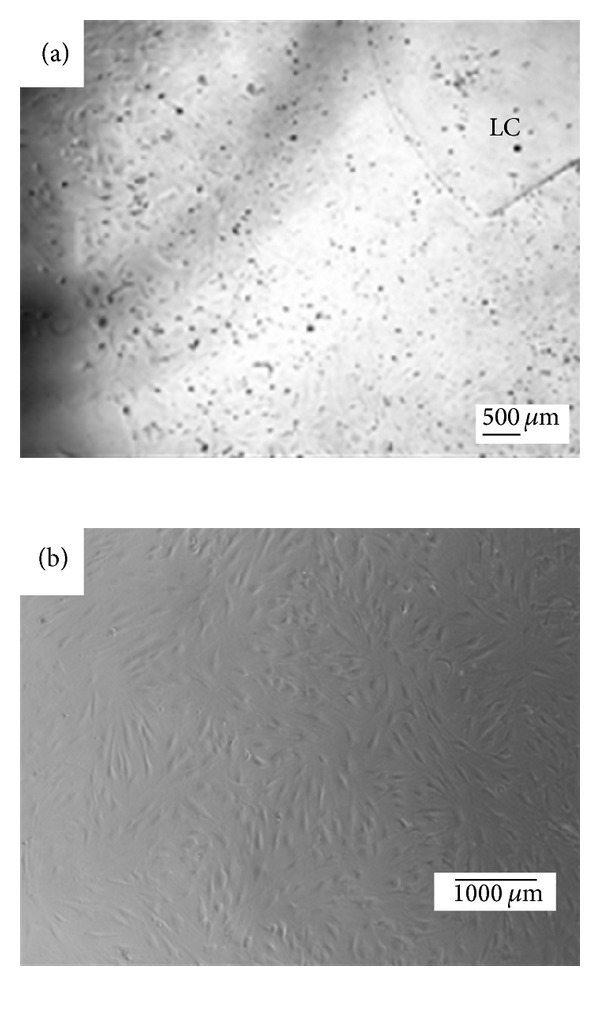
Examples of attached human eye explants with the growing cells: (a) anterior lens capsule (aLC) in a 12-well plate; (b) fibrovascular epiretinal membrane (fvERM) cells growing attached to and independently from the tissue explant in a cell culture Petri dish.

The cells started proliferating out of the explants in 2-3 days (Figure 2). The method for attachment of human eye tissue explants to the 12-well plates is shown in Figure 2(a)—the aLC explant and the cells are flattened under the gravitational force of the viscoelastic material. The fvERM cells grew out of the explants within 24 hours and continued proliferating independently throughout the study period (for more than 6 months) (Figure 2(b)).

### 3.2. Mechanical Stimulation and ACh Induce Rise in [Ca^2+^]_i_ in the aLC-LECs

**Figure 3:**
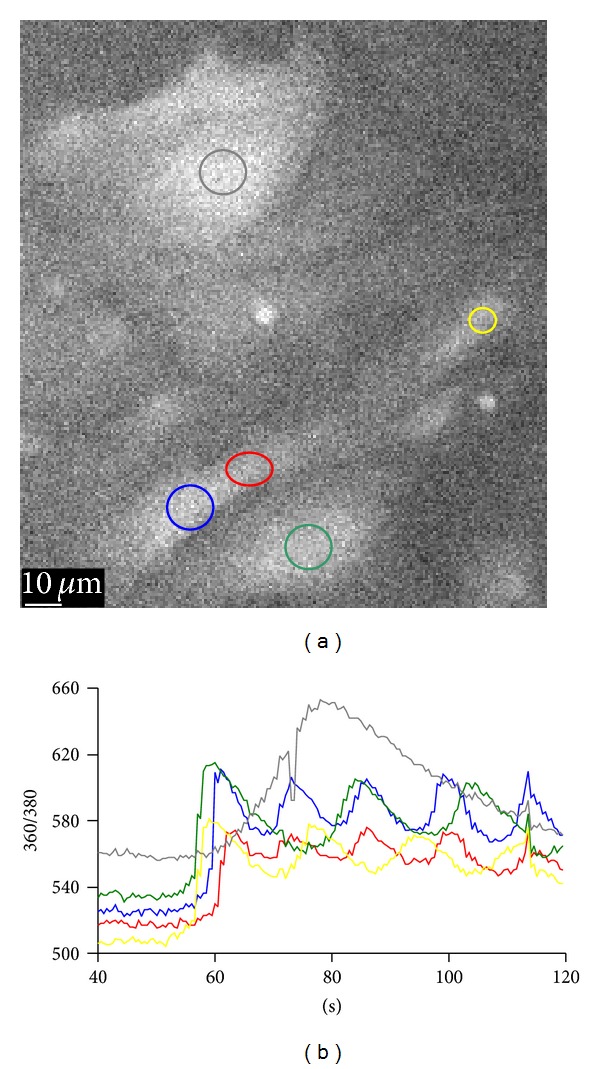
Calcium signaling upon agonist ACh stimulation of the aLC explant-cultured cells (a). The traces (b) represent the time courses of the 360/380 ratio (*R*), proportional to [Ca^2+^]_i_ and correspond to the regions of interest (ROI) shown on the B&W image (a) in the same colors (explant growth time: 28 days).

The functionality of the aLC-LECs attached under the viscoelastic was examined during mechanical stimulation and application of agonist ACh, both of which induced rise in the [Ca^2+^]_i_. Representative examples of 6 explant cultures were analyzed for mechanical stimulation containing 27 cells being stimulated (mostly the cells on the glass surface and some on the aLC); similarly, representative examples of 5 explant cultures were analyzed for ACh stimulation.  Figure 3 shows the calcium signaling upon agonist ACh stimulation of the aLC explant-cultured cells. The oscillations of [Ca^2+^]_i_ are clearly visible here, with each cell having its own frequency of oscillation (Figure 3(b), upper part): 50 cells were analyzed here, out of which 15 (30%) had oscillating response with average of 16.6 ± 4.4 sec from minimum to minimum. Accommodation can be observed for the green trace as the interval between the two maxima decreases with time, while a time delay of 2-3 sec in the [Ca^2+^]_i_ propagation can be seen (Figure 3(b), lower part) needed for the [Ca^2+^]_i_ to reach its first maximum for different ROIs of the same cell (blue and red). No mechanically stimulated cell responded at oscillation manner. The transient responses to mechanical stimulation were usually comparable to those elicited by ACh.

**Figure 4:**
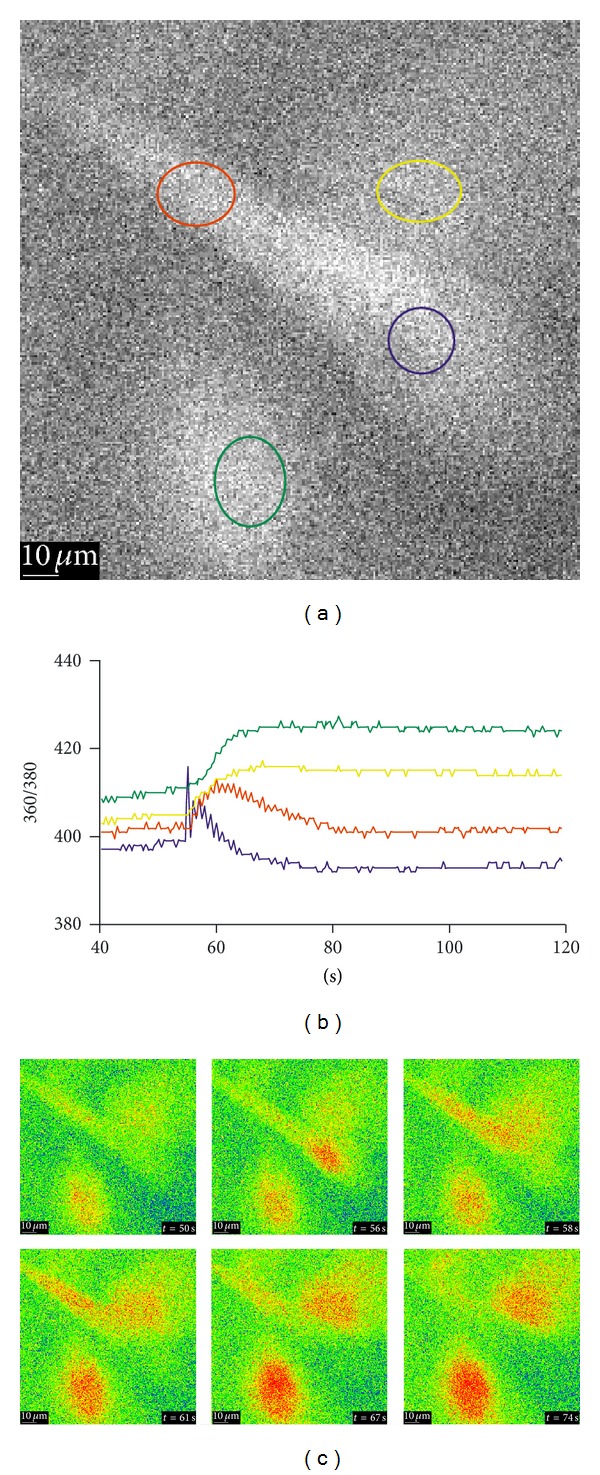
Calcium signaling upon mechanical stimulation of a single cell of the aLC explant culture, showing the [Ca^2+^]_i_ propagation and involvement of intercellular connections. The traces (b) represent the time courses of the 360/380 ratio (*R*), proportional to [Ca^2+^]_i_ and correspond to the regions of interest (ROI) shown in the B&W image (a) in the same colors. (c) A series of the 360/380 ratio images at the time points are indicated. The values for *R* are color coded with blue/green representing low ratio values and yellow/red representing high ratios (explant growth time: 14 days).

The calcium signaling upon mechanical stimulation of a single cell of the aLC explant culture showed [Ca^2+^]_i_ propagation as well (Figure 4)—in the example shown, 2/6 cells had response with two peaks, the first one being bigger than the other and the time interval between the peak maxima being 25 and 26 sec; the rest of the cells had no or very small calcium increase. The blue ROI represents the stimulation site and the red ROI represents the more distal site. There is a delay of around 5 sec in the time needed for the [Ca^2+^]_i_ to reach its maximum at two selected ROIs. The increases in [Ca^2+^]_i_ in the cells surrounding the mechanically stimulated cell suggest the involvement of intercellular connections.

**Figure 5:**
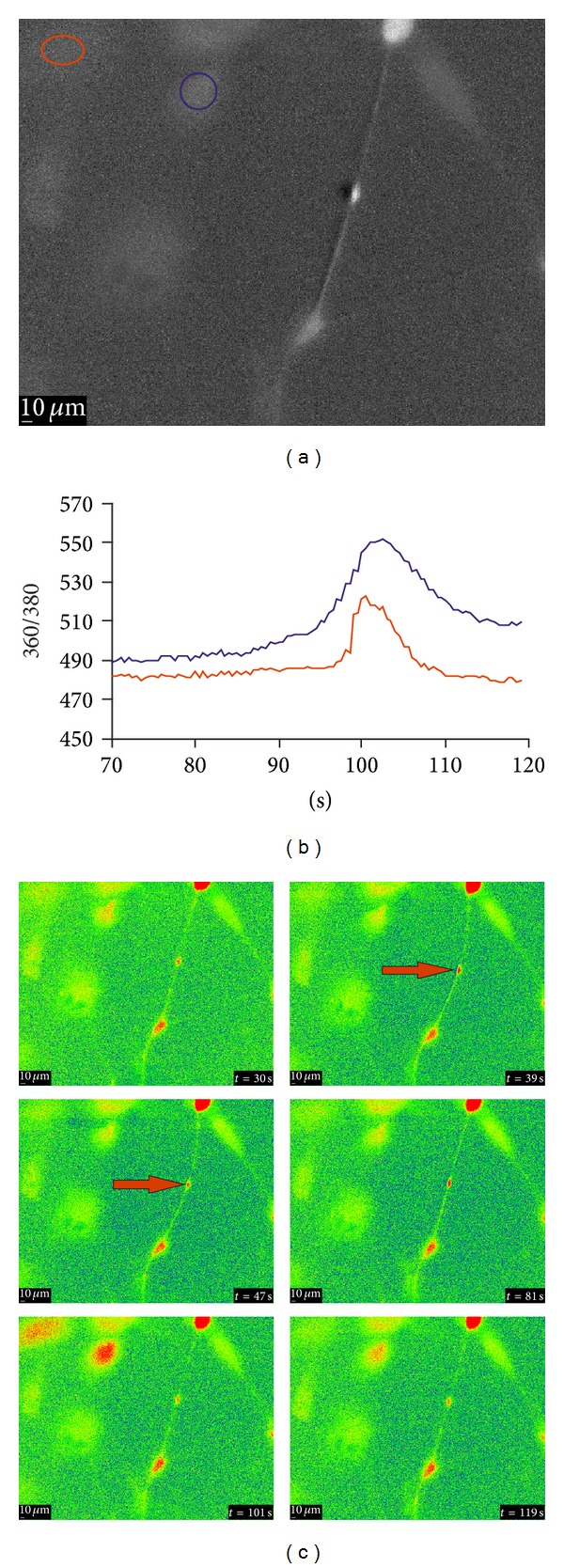
The intercellular dendrite connection strength upon mechanical fluid movement for the nonattached dendrites in aLC explant cultures. The traces (b) represent the time courses of the 360/380 ratio (*R*), proportional to [Ca^2+^]_i_ and correspond to the regions of interest (ROI) shown in the B&W image(a) in the same colors. (c) A series of the 360/380 ratio images at the time points are indicated. The values for *R* are color coded with blue/green representing low ratio values and yellow/red representing high ratios (explant growth time: 21 days).

The intercellular dendrite connection strength upon mechanical fluid movement for the nonattached dendrites in aLC explant culture could also be observed (Figure 5). Indeed, a confirmation that the [Ca^2+^]_i_ changes are not dependent on the mechanical effect of fluid movement but on ACh is shown by the fact that [Ca^2+^]_i_ increase occurs much later (*t* = 101 s) in comparison to the dendritic movement dependent on the mechanical effect of fluid movement (*t* = 39–47 s) as visible on Figure 5(c).

The [Ca^2+^]_i_ dynamics upon mechanical stimulation of fvERMs has been previously described by our group [5], which is a proof of the viability and functionality of these cells.

### 3.3. Measurement of Proinflammatory/Angiogenic Factors Secreted by the fvERM Outgrowing Cells upon TNF*α* Treatment

**Figure 6:**
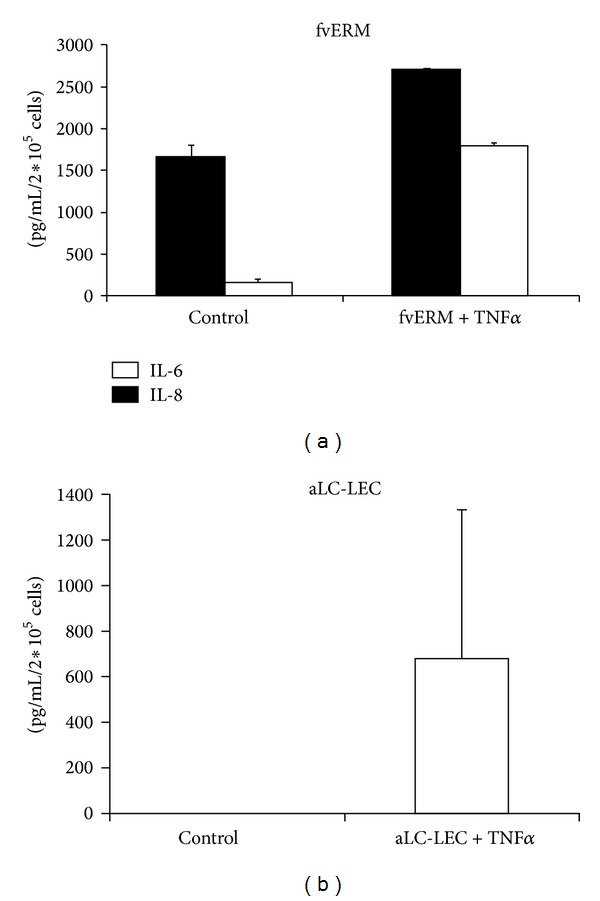
Cytokine secretion by aLC-LEC and fvERM outgrowing cells upon TNF*α* treatment.

The outgrowing cells from the fvERMs showed basal expression of the proinflammatory cytokine interleukin- (IL-) 6* ex vivo*, which was further enhanced by TNFα stimulation. Similar enhancement was noted in the proinflammatory cytokine release of IL-8 upon TNFα stimulation (Figure 6(a)). In the case of aLC-LECs, there were no basal IL-6 and IL-8 responses and TNFα-induced IL-8 secretion (Figure 6(b)).

## 4. Discussion 

A novel, simple, and reproducible method for creating adherent conditions for human eye explants and* ex vivo* cellular expansion using viscoelastic material as well as studies on calcium dynamics and inflammation is established here. The outgrowing cells, over time, migrate out of the explants and grow adherently onto the surface of the cell culture dish, showing signs of continuous proliferation.

Alternative adherence methods for tissues explants can be the use of dry surface, concentrated serum drop, or the fibrin-glue method—the latter being used mostly for* in vivo* purposes. The advantage of the viscoelastic method is in avoiding extreme conditions such as dryness and serum stimulants, yet preserving natural architecture of the tissue and standard nutritional conditions for the cells. The viscoelastic is an inert substance having viscous, elastic, and gravitational properties which force the graft to attach to a surface. The viscoelastic HEALON OVD is used in ophthalmic surgical procedures to maintain deep anterior chamber, which facilitates manipulation inside the eye with reduced trauma to the corneal endothelium and other ocular tissues.

Two tissue types are used here to establish adherent* ex vivo* explant cultures: aLCs containing LECs and fvERMs. Tissue and cell adherence allow measurement of the [Ca^2+^]_i_ upon mechanical or pharmacological stimulation, giving advantage of having less noise from cellular movement within the cell culture dish.

Precise regulation of the [Ca^2+^]_i_ levels is critical for maintaining normal cellular function, fluctuations of which can act as signals for numerous physiological or pathological events. Imbalance in the [Ca^2+^]_i_ levels may lead to development of cataract in the lens [6–9]. Our results indicate an increase in the [Ca^2+^]_i_ upon mechanical stimulation and application of ACh to aLC-LECs. Previously, mechanical stimulation had been used to induce [Ca^2+^]_i_ rise in cultured bovine LECs [10]. Such stimulation of a single cell within a confluent layer was shown to initiate cell-to-cell calcium signaling. Contractions in human aLECs attached to the surgically isolated capsules could also be mechanically induced [11].

The increase in [Ca^2+^]_i_ suggests involvement of intercellular connections between the LECs studied* ex vivo*. In human aLECs, ACh binds to M1 muscarinic receptors (M1 mAChR) and induces a rise in [Ca^2+^]_i_ [12–14]. The origin of ACh in the lens is not clear; however, its presence can certainly affect cells of the immune system, which possess membrane bound mAChR and nicotinic (nAChR) receptors that can regulate their function [15–18]. Choline acetyltransferase (ChAT) enzyme expression in CD4^+^ and CD8^+^ T-cells has been previously shown, suggesting that lymphocytes possess all of the necessary biochemical machinery to produce this neurotransmitter, thereby regulating their function in an autocrine manner [19]. In general, according to the data obtained in mammalian models, it has been proposed that cholinergic activity increases as a result of direct contact between T-cell receptor (TCR)/CD3 molecules, CD4 and CD8 coreceptors, and other accessory molecules [17]. Experimental data obtained by means of* in vitro* models and in absence of neuronal innervation have shown ChAT production in B-cells, macrophages, and dendritic cells from mice; production of this enzyme appears to be upregulated by Toll-like receptor (TLR) activation, a pathway acting via MyD-88 [19]. Moreover, Neumann et al. in 2007 [20] showed in human leukocytes that antagonists of the nicotinic and muscarinic receptors (tubocurarine and atropine, resp.) could significantly decrease the phagocytic functions of granulocytes but did not change the migration of these cells, whereas in Jurkat cells (the human helper T-lymphocyte leukemic line) exposure to oxotremorine-M (Oxo-M), a cholinergic agonist, could significantly increase the synthesis of IL-2, which could be related to the transcriptional factor activator protein-1 (AP-1) and mitogen-activated protein kinases (MAPK) [21]. Experiments with MOLT-3 cells (the human T-cell leukemia line) showed involvement of the protein kinase C (PKC) signaling pathway-MAPK, cyclic adenosine 3′,5′-monophosphate (cAMP), and calcineurin in the synthesis of ACh [18]. There are findings suggesting that photoreceptor outer segments (OS) communicate via neurotransmitters such as ACh and SLURP-1, while RPE cells may receive these signals through nAChRsα7 in their microvilli [22]. It cannot be ruled out, however, that other cells including LECs can be activated by ACh in such a manner. Indeed, evidence that RPE cells can express nAChRs, similar to how other epithelial cells do, has been related to cell development, death, migration, and angiogenesis [23]. The nAChRα7 is responsible for the inhibition of macrophage TNF release via the parasympathetic anti-inflammatory pathway [24], thus opening up new avenues for the design of experimental anti-inflammatory therapeutics in different segments of the eye.

The adherent aLC-LEC cultures can be used for inflammatory studies as well. Inhibition of aldose reductase (AR), for example, can prevent lipopolysaccharide- (LPS-) induced inflammatory response in human LECs [25], such as synthesis of large quantities of bioactive inflammatory mediators: nitric oxide, prostaglandins, TNF-*α*, IL-1, IL-6, and IFN-*γ* [26–28]. Ocular tissues can be exposed to various proinflammatory factors released due to injury, infection, or disease [29–31]. The lens can also be exposed to such factors appearing in the aqueous humor during bacterial infections [32–34]. It was shown that incubation of human LECs with cytokines such as TNFα increases the activation of nuclear factor kappa-light-chain-enhancer of activated B-cells (NF-*κ*B) and causes cytotoxicity [35, 36] as well as apoptosis in human LECs (HLECs). Preventing NF-*κ*B activation by AR inhibitors should therefore rescue HLECs from cell death and inflammation [36, 37]. Transforming growth factor- (TGF-) *α* and TGF-*β* (2), mRNA can also be synthesized by human cataract LECs* in situ*, while IL-8 mRNA can be synthesized* in vitro* [38]. IL-1, IL-6, and basic fibroblast growth factor (b-FGF) can be produced* in vivo* by residual LECs following cataract surgery, which can cause postoperative inflammation and LEC proliferation. IL-1 and TGF*β* may participate in the postoperative inflammation by increasing PGE2 synthesis by residual LECs [39]. The role of these cytokines which can be synthesized by the LECs* in vitro* may, therefore, be significant in studying proliferation of LECs after cataract surgery, which can eventually lead to inflammation and secondary cataract [40]. It was revealed that the expression of TNFα gene in LECs is more extended compared to that of IL-1*α* in lens capsule samples obtained from cataract surgery [35].

Cell death studies using terminal deoxynucleotidyl transferase- (TdT-) mediated dUTP nick-end labeling (TUNEL) of LECs in capsulotomy specimens found necrotic cell death caused by damage during or soon after cataract surgery. Loss of cells from the lens epithelium by apoptosis or other mechanisms of cell death does not seem to play a major role in age-related cataract formation [41].

Proper phenotypization of the cell surface markers of* ex vivo* cultured cells growing out of human fvERMs from PDR gives possibility to study their role and function in immunity. The cell adhesion molecules (CAMs) and integrins profile are meaningful in structuring the cell-based tissue integrity and immune processes. Application of high-throughput screening by angiogenic protein arrays allows measuring the angiogenic potential of fvERM outgrowing cells under presence or absence of proinflammatory factor TNF*α* [5].

Presence of TNF*α* in the vitreous is important marker for PDR [42, 43]. High levels of IL-6, IL-8, and TNF*α* have been measured in the vitreous of PDR patients [2, 3], giving support to the role of inflammatory cytokines in angiogenesis in PDR. Increased secretion of IL-6 and IL-8 was also measured in our fvERM outgrowing cells upon TNF*α* stimulation using the ELISA method. Understanding their role can provide important diagnostic and therapeutic targets for the treatment and prevention of inflammation and angiogenesis in PDR.

Fourteen main TNF*α*-inducible proteins have been reported in the literature in relation to immune response, among them being the pentraxin-related protein 3 (PTX3), a known marker for rapid primary local activation of innate immunity and inflammation. ICAM-1 expression increased upon TNFα proinflammatory stimulus in primary hRPE cells, similar to the fvERM outgrowing cells, giving a link to the function which these activated cells may play in leukocyte adhesion [44–50]. Endothelin 1 (ET-1) molecule secreted by endothelial cells when stimulated by proinflammatory cytokines increases in the vitreous of patients with PDR [51], also detected in the fvERMs upon TNFα treatment in our previous study [5]. IL-1*β* concentrations are higher in the vitreous of patients with PDR compared to non-PDR and controls, indicating that there could be a minimal acute inflammatory activity present in the early stages of retinopathy, which progressively increases in the most advanced stages of the disease. In comparison, the level of IL-1Ra, which is an anti-inflammatory cytokine, was found to be significantly higher in the controls compared to those of PDR [51]. The process of complement C5a activation leads to release of cytokines, reactive oxygen species, proteolytic enzymes, and other proinflammatory molecules [52].

Recently, the extracellular high-mobility group box-1 (HMGB1) was reported as proinflammatory cytokine [53–56] playing a role in angiogenesis [53, 57–59], and it was detected in the vitreous of patients with PDR together with MCP-1 and sICAM-1 [60]. HMGB1 and the soluble receptor for advanced glycation-end products (RAGE) are also expressed by vascular endothelial and stromal cells in fvERM from PDR, suggesting a role for the HMGB1/RAGE signaling axis in the progression of PDR [53, 57–59]. A significantly elevated level of five novel cytokines including sCD40L, GM-CSF, IFNα2, IL-12p40, and MCP-3 in the vitreous of PDR patients previously not associated with the disease was also recently reported [61].

In conclusion, providing adherent, inert conditions for* ex vivo* cultivation and expansion of cells from different tissues is crucial for establishing disease models. Using viscoelastic material as a novel and simple method for achieving tissue and cell adherence can empower studies on intracellular calcium dynamics upon mechanical stimulation, calcium signaling, and intercellular communication upon ACh stimulation as well as inflammatory studies in as little background noise and artifacts as possible, void of detachment-associated cell death and associated inflammation. Future studies on cell functionality and homeostasis using calcium imaging and inflammation screening widen the possibilities for development of pharmacological and cell-based therapies that are attractive approach for treating eye diseases.
